# Family Caregiver Learning Needs Immediately Following a Dementia Diagnosis

**DOI:** 10.1002/alz70858_106828

**Published:** 2025-12-26

**Authors:** Carolyn K Clevenger, Laura J Medders, Molly Perkins, Glenna S Brewster, Fayron Epps, Kenneth Hepburn

**Affiliations:** ^1^ Emory University, Atlanta, GA, USA; ^2^ Emory Healthcare, Atlanta, GA, USA; ^3^ Emory University, Nell Hodgson Woodruff School of Nursing, Atlanta, GA, USA; ^4^ UT Health San Antonio, San Antonio, TX, USA

## Abstract

**Background:**

Family caregivers navigate and manage a variety of family, medical, financial, social services, and legal concerns for the person living with dementia. How caregivers of persons living with dementia develop and strengthen these essential navigational skills has been largely overlooked.

**Method:**

As part of a larger study, we engaged 15 participants in three focus groups to inform the course revisions of a previously piloted version. Each group was presented with a content outline of the proposed educational intervention and invited to suggest exclusion or inclusion of topics based on their experiences as caregivers. Groups were facilitated by two experienced qualitative researchers and these sessions were recorded and transcribed verbatim. We used a thematic analysis approach to analyze the data. Two primary coders (the qualitative interviewers) coded each transcript independently and met to reconcile any coding differences. Final themes were fully developed and confirmed based on input from the full research team.

**Result:**

Most participants identified as female (73.3%) and were actively caregiving. These were, typically, spouses (40%) or adult children (33.3%) Participants appreciated the amount of detailed information the draft course outline provided regarding navigating family, medical, financial, social services and legal systems but recommended several ways for strengthening this material. They highlighted the need to better emphasize material regarding choice‐making and timing (e.g., the need to be proactive and make decisions while care recipients could still participate in the decision‐making process) and shared strategies they had used successfully or not so successfully when interacting with these systems. Participants also stressed the importance of self‐care and recommended several ways for avoiding becoming overwhelmed and overburdened. Some participants identified stigma associated with dementia and cultural values for privacy as key barriers to effectively navigating this “journey.”

**Conclusion:**

While we found that topics we outlined based on a previously piloted psychoeducational program for dementia family caregivers provided salient information for caregivers, feedback from focus group participants indicated several ways this material could be strengthened to better guide these stakeholders in navigating their caregiving journey. These findings have been key to informing the newly launched *Caregiver as Navigator* intervention.

